# Our New York Letter

**Published:** 1891-08

**Authors:** 


### OUR NEW YORK LETTER.

NEW YORK, July 20, 1891.

Dr. T. McGilliandy, Instructor of Obstetrics at the New York Polyclinic, at a recent meeting of the New York County Medical Association, read an extremely interesting paper on "Anomalies of the Breast," and exhibited ten cases, thus increasing the number reported in American medical literature from fifteen to twenty-five. Among the patients exhibited by him was one, a male, with a large breast situated on the posterior portion of the thigh. Another was an infant, seven weeks old, with three nipples in a row; and still another, a woman, with four breasts, in the axillary region, two of them with a well marked areola, but no nipples.

The author, in speakiug of the treatment of this class of cases, said that they did not call for operative interference of any kind, inasmuch as the presence of the anomalies gave rise to no trouble other than a slight inconvenience during the time the gland was secreting.

The Medical department of the University of the City of New York makes a special announcement this year which is of general interest to the profession throughout the country. This announcement indicates another great advance in educational matters. The Faculty have placed the college on a true university basis. Its pecuniary interests have been put altogether in the hands of a body separate from the faculty, while the latter receive fixed salaries, are free to give their time and ability to advance the standard of medical education, unincumbered by financial cares. This step, as it can be readily understood, renders the faculty wholly independent of the number of students who may attend the college classes.

Another change, indicative of progress with this institution, consists in making a three years' course obligatory with all matriculants. In addition to these two steps in the right direction, a third one is the introduction of a course of recitation in place of the didactic lectures during the first and part of the second year.

It is to be hoped that the improvement which has thus began in the matter of raising the standard of medical education may continue until it has spread its healthy influences throughout other parts of the country.

Dr. Florian Krug, of this city, read a very excellent paper before the Obstetric Section of the New York Academy of Medicine, entitled, "My Personal Experience with Vaginal Hysterectomy," in which he stated that his own experience, based on fifteen vaginal hysterectomies, done during the last three years, controverts the objections raised by the adversaries of vaginal hysterectomy. Out of fourteen cases in which he used ligatures exclusively in tying the ligaments, he has not lost a single case. The only case that terminated fatally was one in which he used forcipressure, the patient dying on the sixth day from septic peritonitis. Judging, therefore, from his own experience, as well as the experience of the German operators, he felt constrained to say that vaginal hysterectomy is not a dangerous operation.

The first important point in its performance is to have the field of operation absolutely aseptic. The patient is first anesthetized, and then the Paquelin cautery is freely applied, and for about a week or so frequent vaginal douches are given; sometimes tannic acid and iodoform powder are applied until finally a clean looking surface is obtained. During this preparatory treatment the management of the bowels receives special attention.

Directly after the operation the vagina is thoroughly scrubbed with mollein, containing ten per cent. of creolin, by means of a brush. After this a disinfectant douche of bichloride is given.

A second important point is the care of the stumps after extirpation. In order to secure perfect safety they must be treated extra-peritoneally; in other words, their raw surfaces must be prevented from coming in contact with the intestines. By the use of ligatures this object can be easily accomplished by slightly inverting the stump towards the vagina and carefully packing the peritoneal wound with iodoform gauze. If ligatures be used, and proper asepsis be maintained during the operation, the iodoform gauze may be left in position for eight days, when on changing it the peritoneal cavity will be found well closed by healthy granulations.

The wide difference in the results of European and American operators in cases of vaginal hysterectomy, Dr. Krug thinks, can only be explained by the fact that abroad the patient generally comes under the care of the surgeon at an early date, while in this country the surgeon sees the patient only when the disease has advanced to a stage when nothing known to science can save her from her terrible fate.

Dr. Polk, in discussing the paper, said that it seemed to him within certain limitations the operation of vaginal hysterectomy was the only one to do. He had operated for uterine cancer twenty-two times with a mortality of five; three were cases that should not have been operated upon, and in two, he attributed the cause of death to his imperfect operative technique.

Dr. Janvrin said it had been his custom to never operate unless the disease was confined to the cervix or body of the uterus. If he had any idea that the disease had extended beyond these parts he would not subject his patient to the risk of a vaginal hysterectomy. He had performed in all eleven operations, with the following results. In the first two cases he used the ligature, in seven clamps, and in the last two the ligature. One death occurred among the first two cases ; another patient died within the past year from septic peritonitis. Of the nine which recovered he had lost sight of only three. The first operation dates back about a year, and the last about four weeks.

Dr. B. McE. Emmet said that he had only operated in four cases of vaginal hysterectomy, with one death from shock twenty-four hours after the operation. He used the clamp in all cases, and with great satisfaction. He thought the dangers of the clamps came from their being dull.

